# Induction of G1-phase cell cycle arrest and apoptosis pathway in MDA-MB-231 human breast cancer cells by sulfated polysaccharide extracted from *Laurencia papillosa*

**DOI:** 10.1186/s12935-016-0315-4

**Published:** 2016-05-26

**Authors:** Hossam Murad, Mohammad Hawat, Adnan Ekhtiar, Abdulmunim AlJapawe, Assef Abbas, Hussein Darwish, Oula Sbenati, Ahmed Ghannam

**Affiliations:** Division of Human Genetics, Department of Molecular Biology and Biotechnology, AECS, P. O. Box 6091, Damascus, Syria; Division of Biochemistry & Toxicology, Department of Molecular Biology and Biotechnology, Damascus, Syria; Division of Mammalian Biology, Department of Molecular Biology and Biotechnology, Damascus, Syria; Laboratory of Marine biology, Faculty of Sciences, Tishreen University, Lattakia, Syria; Laboratory of plant functional genomics, Department of Molecular Biology and Biotechnology, AECS, P. O. Box 6091, Damascus, Syria

**Keywords:** G1-phase cell cycle arrest, Apoptosis, MDA-MB-231, Red algae

## Abstract

**Background:**

Marine algae consumption is linked to law cancer incidences in countries that traditionally consume marine products. Hence, Phytochemicals are considered as potential chemo-preventive and chemotherapeutic agents against cancer. We investigated the effects of the algal sulfated polysaccharide extract (ASPE) from the red marine alga *L. papillosa* on MDA-MB-231 human breast cancer cell line.

**Methods:**

Flow cytometry analysis was performed to study the cell viability, cell cycle arrest and apoptosis. Changes in the expression of certain genes associated with cell cycle regulation was conducted by PCR real time analyses. Further investigations on apoptotic molecules was performed by ROS measurement and protein profiling.

**Results:**

ASPE at low doses (10 µg/ml), inhibited cell proliferation, and arrested proliferating MDA-MB-231 cells at G1-phase. However, higher doses (50 µg/ml), triggered apoptosis in those cells. The low dose of ASPE also caused up-regulation of *Cip1/p21* and *Kip1/p27* and down-regulation of cyclins *D1*, *D2*, and *E1* transcripts and their related cyclin dependent kinases: *Cdk2*, *Cdk4*, and *Cdk6*. The higher doses of ASPE initiated a dose-dependent apoptotic death in MDA-MB-231 by induction of Bax transcripts, inhibition of Bcl-2 and cleavage of Caspase-3 protein. Over-generation of reactive oxygen species (ROS) were also observed in MDA-MB-231 treated cells.

**Conclusions:**

These findings indicated that ASPE induces G1-phase arrest and apoptosis in MDA-MB-231 cells. ASPE may serve as a potential therapeutic agent for breast cancer.

## Background

Marine algae are health-enhancing resource for providing nutritional benefits and helping diseases treatment. Several epidemiological studies provided evidence that marine algae consumption correlates with low breast cancer rates in East-Asia. These studies report that low risks of developing breast cancer is associated with seaweeds intake in diet [1, 2]. The potentially beneficial effects of marine algae are partially attributed to polysaccharide compounds, particularly sulfated ones [3–5]. Carrageenans are a family of linear sulfated polysaccharide (SP), extracted mainly from red algae [6–8]. Depending on their sulfation degree, solubility and gelling properties, three categories of carrageenans have been categorized as kappa-, iota- and lambda-carrageenans [9, 10]. Red algae (*Rhodophyta*) have been documented as a source of natural nutraceuticals and pharmaceuticals for many years. Recent studies showed that sulfated polysaccharides isolated from red seaweed possesses various therapeutic and biological feature such as anti-oxidants [11], anti-proliferative, anti-tumor [12–16], anti-viral [10, 17] and anti-coagulants [18, 19].

Breast cancer is the most common cancer as well as the second leading cause of cancer-related deaths in women across the world. One out of ten women over 55 years of age is frequently diagnosed with breast cancer [20]. Dietary pattern has been identified as one of the major factors for the difference in breast cancer incidence [21]. Major issues concerning conventional anti-cancer chemotherapy are the occurrence of side effects induced by the non-specific targeting of both normal and cancerous cells [22, 23]. Based on this, there has been growing interest in the use of naturally occurring molecules with chemo-preventive and chemotherapeutic properties in cancer treatment [3]. Natural products will thus continue to play major role as active substances, model molecules for the discovery and validation of new drug targets [24].

Breast cancer cell lines are useful tools for studying the mechanism of new nutraceuticals, pharmaceuticals and drugs effects on mammalian cells. MDA-MB-231 cell line is a human breast cancer cell line known to be widely used in such studies. A previous study indicated ASPE preparation from the red alga *Laurencia papillosa.* This ASPE predominantly contains a sulfated polysaccharide. ASPE could inhibit proliferation of MDA-MB-231 in vitro in a time and dose dependent manner [25]. However, the anti-proliferative activity mechanism of ASPE remains unclear. The purpose of the present study was to elucidate the mechanism of ASPE anti-proliferative effect. Furthermore, it was to characterize the cell cycle arrest and apoptosis induced in MDA-MB-231 cells after ASPE treatment.

## Methods

### Plant material collection and preparation of polysaccharide extract

Red alga *L. papillosa* was collected from Syrian coastal waters and processed at the marine biology laboratory (Faculty of biological sciences, Tishreen University, Syria). ASPE was prepared as previously explained in [25]. Briefly, collected algal biomass was washed with tap water to remove salt, sand and foreign matter, air-dried to constant weight at 60 °C then heated with water (1.5 % w/v) for 12 h with mechanical stirring. Polysaccharides then dissolved in MilliQ water and filtrated using cheesecloth and immediately mixed with 3 volumes of ethanol (95 %) (Sigma-Aldrich, Germany). This step caused precipitation of polysaccharides which were collected and oven-dried at 50–60 °C to constant weight.

### Cell culture

MDA-MB-231 breast cancer cell line was kindly provided by prof. P. BÉCUWE, Cancer Research Unit (EA SIGRETO), Nancy, France. MDA-MB-231 cells were cultured in RPMI-1640 medium containing 10 % fetal bovine serum (FBS), 50 U/ml penicillin/streptomycin, and 2 m M l-glutamine. Cells were treated with ASPE solved in water for the desired concentrations and times and proceeded for analysis as described below.

### Cell viability assay

TO/PI double staining assay was used to distinguish dead cells from viable ones. 1 × 10^5^ MDA-MB-231 cells were grown for 24 h (hours) then treated with different concentrations of ASPE (5, 10, 50 and 100 µg/mL) or untreated (control), and incubated for another 24 h. Cell viability was estimated by adding 1 ml of viability buffer to labeled 6 ml tubes equals the number of samples to be analyzed. A 20–100 μl of each harvested cell suspension (~1 × 10^5^ cells) were transferred to the matching tubes. A 4 µl of Thiazole Orange (TO) solution (final concentration 1 µg/ml) and 2 µl of propidium iodide (PI) solution (final concentration 2 µg/ml) were added to each tube and incubated at room temperature for 5 min and analyzed directly on already set BD FACSCalibur flow cytometr.

### DNA content/cell cycle analysis

Samples of untreated or treated MDA-MB-231 cell cutlers were analyzed for DNA content/cell cycle analysis by flow cytometry. Cell cycle distribution was calculated after appropriate gating of cell populations in FL-2-Area vs FL-2-Width plot of PI fluorescence. Assays were carried out in triplicates, and the results are representative of three independent experiments.

### Real-time PCR array of human cell cycle related genes

Cells were seeded at 1 × 10^6^ cells and grown for 24 h, then treated with ASPE at two different concentrations: 10 and 30 µg/mL for 24 h. Total RNA was extracted by RN easy kit (Qiagen, Hilden, Germany) and cDNA was synthesized as previously described [25]. For quantitative determination of transcripts of cell cycle pathway, cDNA was mixed with RT^2^ SYBR Green ROX qPCR Master mix (SA Biosciences, USA) according to the manufacturer’s instructions. The expression of 84 genes was assessed using the Profiler™ PCR Array Human Cell Cycle (PAHS-020ZC-12, SA Biosciences, USA) according to the manufacturer’s instructions. Thermal cycling and fluorescence detection were performed using StepOnePlus™ Real-time PCR system (Applied Biosystems, Foster City, CA—USA). Data were analyzed by PCR array data analysis web portal (http://www.sabioscience.com/pcr/arrayanalysis.php), using 2^−ΔΔCt^ method.

### Apoptosis assay

1 × 10^5^ MDA-MB-231 cells were seeded, and treated with ASPE with different concentrations: 10, 25 and 50 µg/mL. Untreated control was also included. Cells then incubated for 24 h. Cell death was evaluated by the loss of membrane integrity (high PI fluorescence) after treatment with PI solution. Phosphatidylserine exposure was determined using Annexin V-FITC/PI double staining kit (BD Biosciences, USA) and analyzed by flow cytometry. Analysis of stained cells can distinguish cells into four groups, namely viable (annexin V^−^ PI^−^), early apoptotic (annexin V^+^ PI^−^), late apoptotic (annexin V^+^ PI^+^) and necrotic (annexin V^−^ PI^+^) cells.

### Flow cytometry protein expression analysis

Cells were treated with ASPE for 24 h and then trypsinized and centrifuged for 3 min at 130×*g*. The cells were re-suspended and washed with PBS. Active Caspase-3 and Bcl-2 proteins expression were evaluated by fluorochromes conjugated anti-bodies. For each protein a 100 μl of cell suspension (~1 × 10^5^ cells) were transferred to 5 ml tube. Cells washed twice with PBS containing 1 % FCS and 0.1 % NaN3 then cells were suspended in 250 µl of 1X fixation/permeabilization buffer, and incubated in dark at 4 °C for 20 min. The cells washed twice with 1 ml of 1X permeabilization and washing buffer, and suspended in 100 perm/wash buffer. 20–30 μl of the fluorochrome conjugated anti-body was added and cells incubated on ice in the dark for 30 min. Then cells washed with 1 ml of PBS containing 1 % FCS and 0.1 % NaN3 and suspended in 500 µl of PBS. Appropriate isotype and autofluorescence controls were also included.

### Measurement of released ROS

Mitochondrial function disorder is often associated with ROS release enhancement. The Amplex Red™, one kind of non-marking and oxidation-sensitive fluorescent probe, which detects the extracellular ROS released by the cell. This protocol was adapted for the measurement of total released Hydrogen peroxide (H_2_O_2_) by lysing cells to detect the both intra- and extracellular produced H_2_O_2_ after ASPE treatment. Cells were seeded at 2 × 10^3^ in 96-well plate before treatment then were treated with different concentrations of ASPE and incubated for 12 h. Cells were washed once with pre-warmed 1 % triton added-sodium pyrophosphate buffer (25–200 mg/L) to prevent H_2_O_2_ degradation when cells lyses, then phosphate buffer containing the Amplex Red™ reagent was applied to the cells according to manufacturer’s instruction (Invitrogen, Carlsbad, USA). Plates were incubated for 15 min at 37 °C. Resorufin, the fluorescent product, was measured in triplicate by a fluorescence multi-well plate reader with an excitation wavelength of 535 nm and an emission wavelength of 590 nm. The values were standardized with reference standard curve of H_2_O_2_.

### Statistical analysis

The results were expressed as the mean value ±SEM of individual experiments. Comparisons of means were conducted using a one-way ANOVA followed by Bonferroni’s post hoc test (GraphPad Prism-Version 6.0 for windows). We considered the two means significantly different when the P value associated was weaker than 0.05.

## Results

### Declines in MDA-MB-231 cell viability and cell death induction following ASPE exposure

**Fig. 1:**
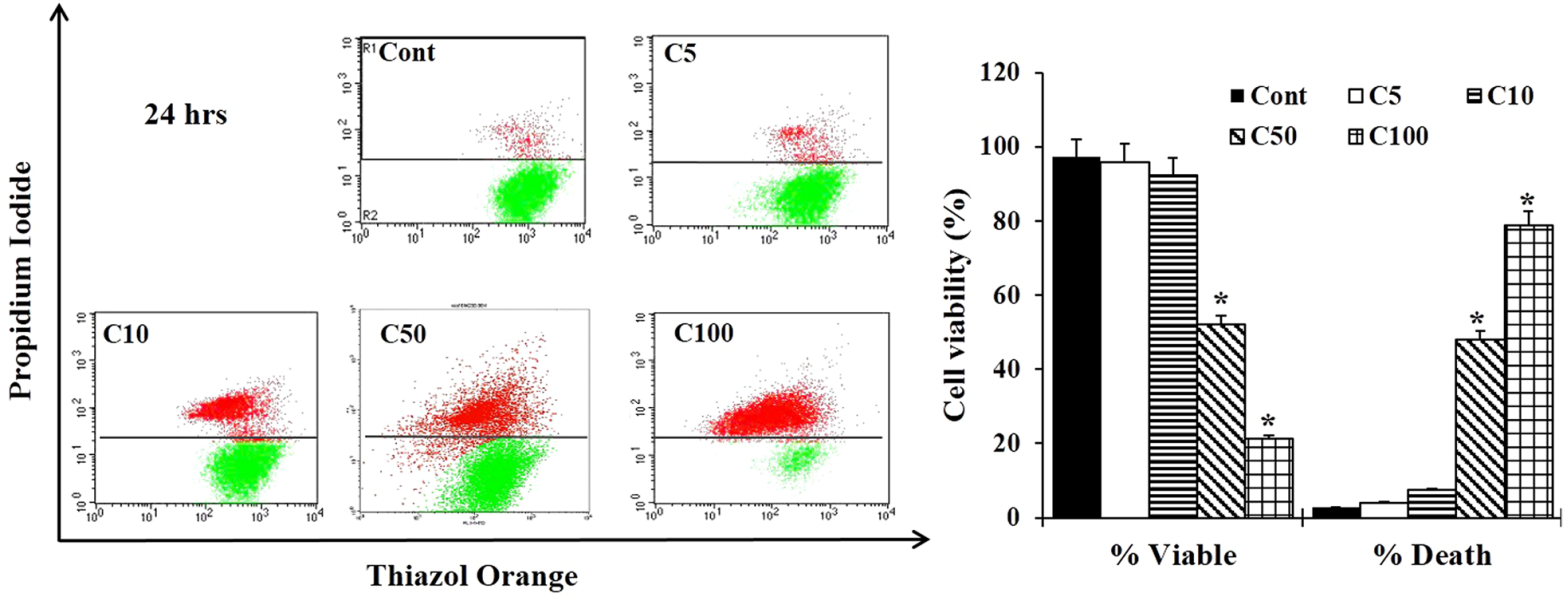
Cytostatic effect of ASPE on MDA-MB-231 cells. Cell viability was determined by flow cytometry analysis after 24 h of ASPE treatment. Untreated (Cont), treated cells with different concentrations: 5 µg/mL (C5), 10 µg/mL (C10), 50 µg/mL (C50) and 100 µg/mL (C100). Viable and dead cells are expressed as a percentage of total cells of the control with each* data point* is representing the mean (±SEM) of three independent experiments. *Asterisks* indicate significant difference (*P* < 0.05)

In this study, the design of experiments focused firstly on investigating the response of ASPE-treated MDA-MB-231 cells using different concentrations. The reduction in viability of treated cells was either due to the ASPE-induced cell death or may attribute to the inhibition of biological or biochemical function in cells exposed to ASPE. Our results showed insignificancy in dead cells in each of the cells exposed to 5 and 10 µg/mL (Fig. 1). In contrast, at a concentration of 50 µg/mL, the number of dead cells increased significantly to reach about 52 % at 24 h of exposure. By augmenting ASPE concentration, the number of dead cells doubled to reach almost 79 % of the population when cells were treated with 100 µg/mL (Fig. 1). Thus, ASPE seems to be capable of exerting a cytotoxic effect on MDA-MB-231 cells under the present experimental conditions.

### G1-phase cell cycle arrest in MDA-MB-231 cells following ASPE exposure

**Fig. 2:**
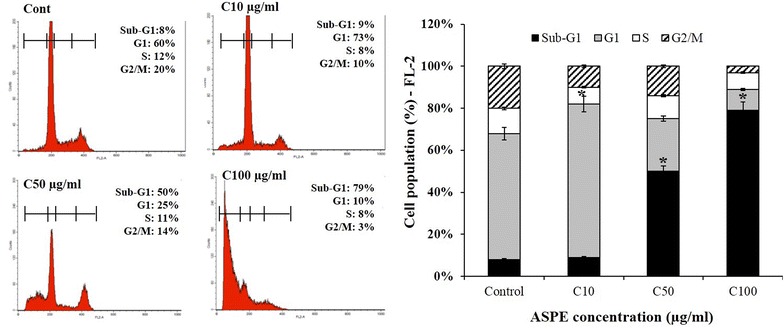
ASPEeffect on the cell cycle distribution in MDA-MB-231 cell. Treated MDA-MB-231 cells with different concentrations of ASPE for 24 h were stained with PI to analyze the cell cycle distribution of each cell type by flow cytometry. Analysis of cell number % of each cell cycle phase relative to total phases. For example, sub-G1 % is calculated as the percentage of the number of cells in the sub-G1 population relative to the number of total cells. Untreated was indicated as (Cont) and treated cells were indicated as 10 µg/mL (C10), 50 µg/mL (C50) and 100 µg/mL (C100). Each* data point* represents the mean (±SEM) of three independent experiments. *Asterisks* indicate significant difference (*P* < 0.05)

ASPE-treated cells with different concentrations 10, 50 and 100 µg/mL for 24 h showed a typical DNA pattern that represented sub-G1, G1, S, and G2/M phases of the cell cycle. Treated cells firstly showed higher G1 population (73 %) compared with 60 % in the control when treated with 10 µg/mL ASPE. This treatment caused a concomitant decrease in the proportion of cells in G2/M phase of the cell cycle from control (20 %) to treated MDA-MB-231 cells (10 %) (Fig. 2). Whereas, the percentages of sub-G1 phase (apoptotic cells) were significantly increased after cells were treated with 50 and 100 µg/mL ASPE up to 50 and 79 % respectively compared with 8 % in the control (Fig. 2). This experiment suggested that ASPE induces G1-phase cell cycle arrest at low concentration (10 µg/mL). Consequently, ASPE treatment at higher concentrations (50 µg/mL) induced cell death in MDA-MB-231 cells.

### Effect of ASPE on the expression levels of cell cycle regulatory genes in MDA-MB-231 cells

**Fig. 3:**
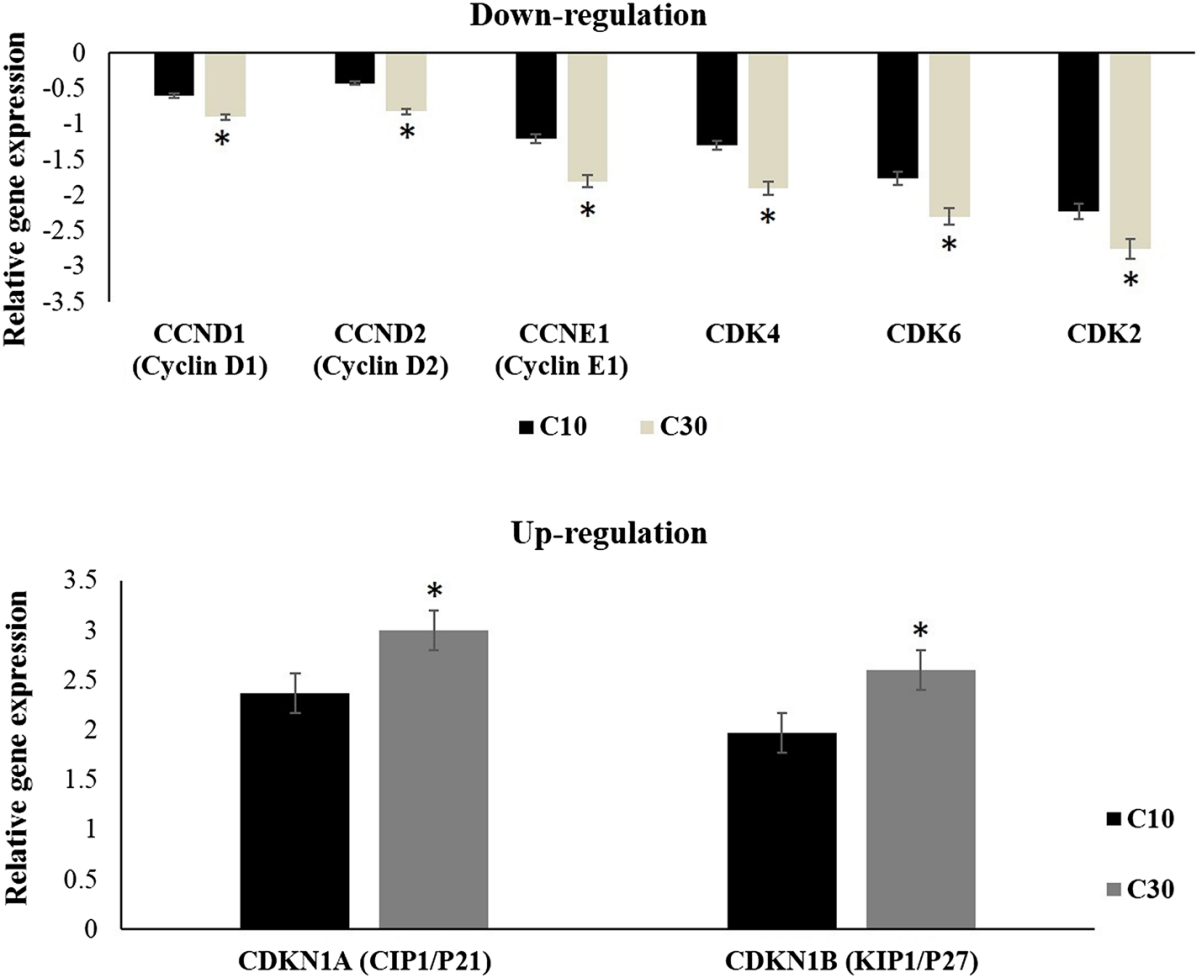
Expression analysis of *cyclin/Cdks* and *Cdks inhibitory* (*Ckis*) genes in ASPE-treated MDA-MB-231 cells. Cells were exposed to 10 and 30 µg/mL of ASPE for 24 h. Change in gene expression in MDA-MB-231 cells of tested genes is mentioned in diagrams as fold change (ratio of target/reference gene). The results were divided in three groups: *cyclin* gene, cyclin-dependent kinase genes (*Cdks*) and cyclin-dependent kinase inhibitory genes (*Ckis*). Each* data point* represents the mean (±SEM) of three independent experiments. *Asterisks* indicate significant difference (*P* < 0.05)

Meanwhile, we revealed ASPE injured DNA of MDA-MB-231 cells and probably triggered the observed G1-phase cell cycle arrest by ASPE. We investigated the effect of ASPE on the expression of cell cycle regulatory genes (*cyclins*, *cyclin*-*dependent kinases* “*CDKs*” and *CDKs inhibitor*). Treatment with ASPE resulted in a clear down-regulation in the gene expression levels of *cyclin D1*, *cyclinD2* and *cyclin E1* at 10 and 30 µg/mL (Fig. 3a). Similarly, a marked decrease in the expression of *CDK2, CDK4* and *CDK6* was detected at 24 h (Fig. 3a). Concomitantly, a significant increase in the expression of *CDK inhibitory* genes (*Cip1/p21* and*Kip1/p27*) was observed (Fig. 3b). These results indicate that ASPE induced at low concentration the CDK inhibitors which play a central role in the cell cycle progression and induced G1-phase arrest of in MDA-MB-231 cells.

### ASPE promotes apoptosis in MDA-MB-231 cells

**Fig. 4:**
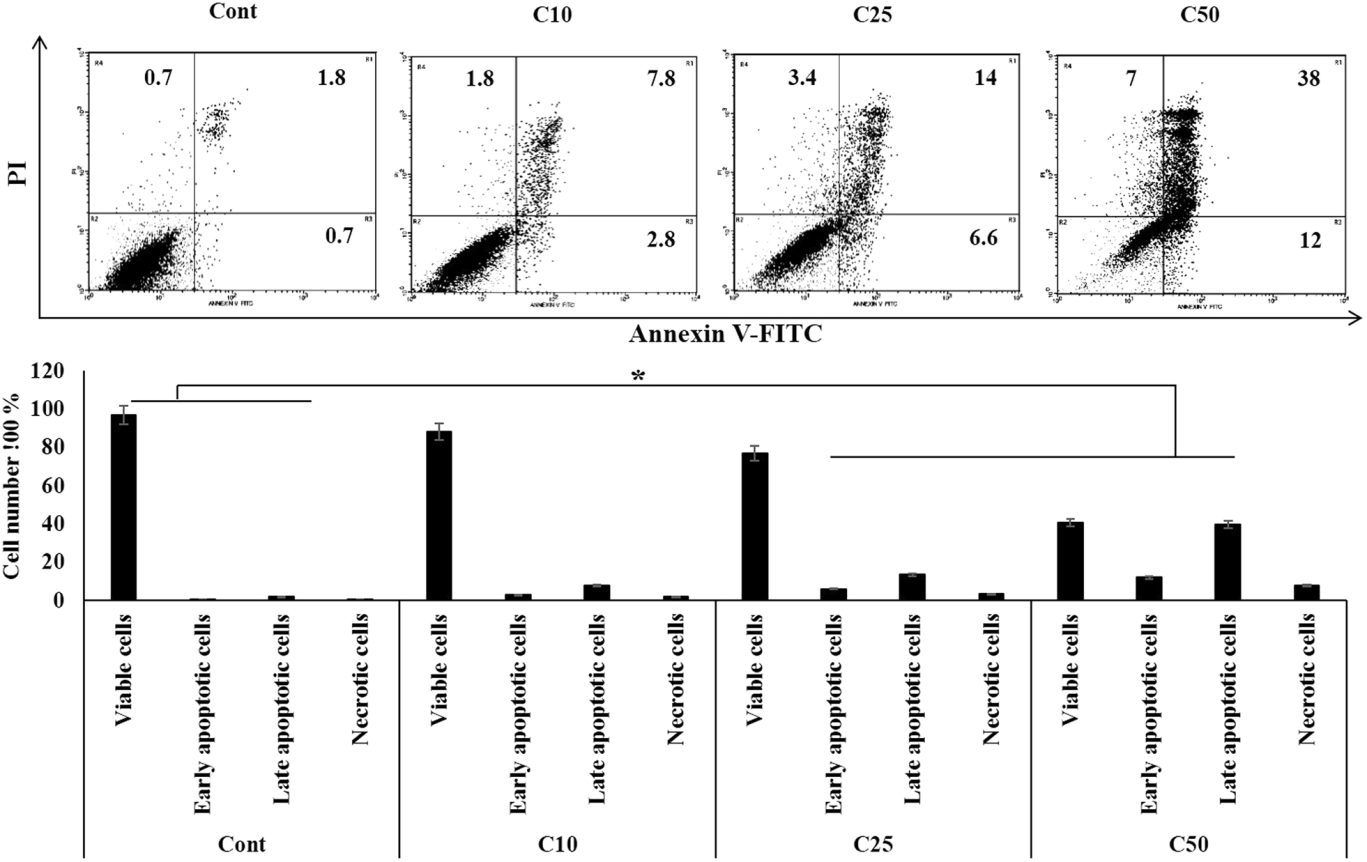
ASPE triggers apoptosis in MDA-MB-231 cells. Flow cytometric Annexin-V/PI binding profiles of untreated (Cont) or treated cells with ASPE at concentrations 10 µg/mL (C10), 25 µg/mL (C25) and 50 µg/mL (C50) for 24 h. Each* data point* represents the mean (±SEM) of three independent experiments. *Asterisks* indicate significant difference (*P* < 0.05)

MDA-MB-231 cells were treated with different concentrations (10, 25 and 50 µg/mL) of ASPE for 24 h. Apoptotic cells were determined by flow cytometry using Annexin V-FITC/PI double labeling. As shown in (Fig. 4), the percentage of the apoptotic cells increased significantly in a dose-dependent manner. Apoptotic cells percentages were: 10.6 % at 10 µg/mL, 20.6 % at 25 µg/mL, and 50 % at 50 µg/mL vs. 2.5 % for the control cell cultures. About 7 % of cell population treated with 50 µg/mL of ASPE showed necrotic signs (Fig. 4).

### Apoptosis induction is regulated through the activation of active-Caspase-3 and inhibition of Bcl-2 protein following ASPE exposure

**Fig. 5:**
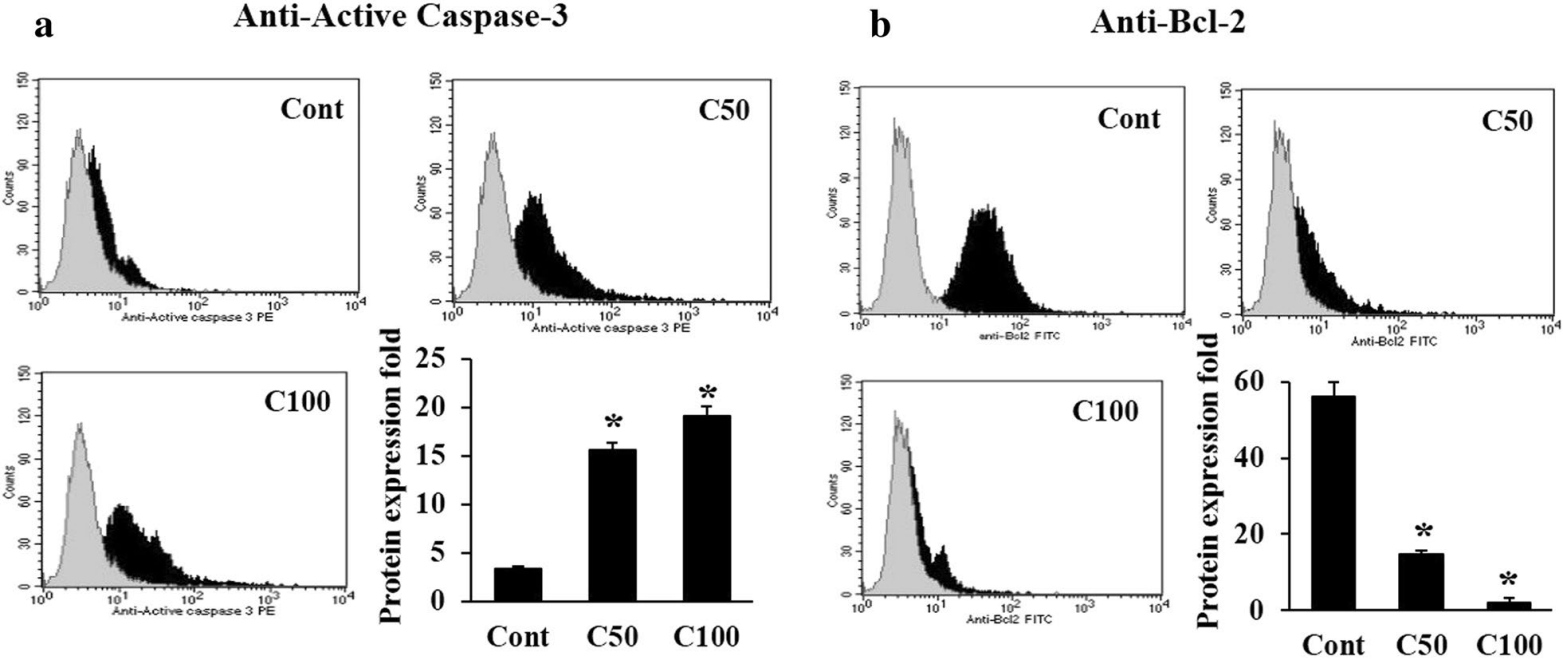
Analysis of apoptotic molecules in treated MDA-MB-231 cells. Flow cytometric expression profile of ACTIVE Caspase-3 (**a**), Bcl-2 (**b**) in MDA-MB-231 cells. Untreated (Cont) or treated with 50 µg/mL (C50) and 100 µg/mL (C100) for 24 h. The* gray* overlapping histogram represents the isotype control. Quantification of expression is expressed as a percentage of total viable cells of the control with each* data point* representing the mean (±SEM) of three independent experiments. *Asterisks* indicate significant difference (*P* < 0.05)

Because ASPE induced a pronounced cell death/apoptosis at higher concentrations (50 µg/mL), we investigated the expression of active-Caspase-3, a crucial protein in apoptosis induction, at 50 and 100 µg/mL to better understand the mechanistic of ASPE signalling. We showed that ASPE induces high levels of active-Caspase-3 protein expression starting significantly at 50 µg/mL ASPE to attend 20-folds of expression change at 100 µg/mL ASPE exposure (Fig. 5a). Thus, these data suggest that Active-Caspase-3 might be involved in the ASPE-induced apoptosis of MDA-MB-231 cells. The down-regulation of Bcl-2 protein at same concentrations was also confirmed by flow cytometry which was decreased in a dose-dependent manner (Fig. 5b).

### ASPE exposure induces disruption in mitochondrial Bax:Bcl-2 ratio and generates the production of reactive oxygen species (ROS) in MDA-MB-231 cells

**Fig. 6:**
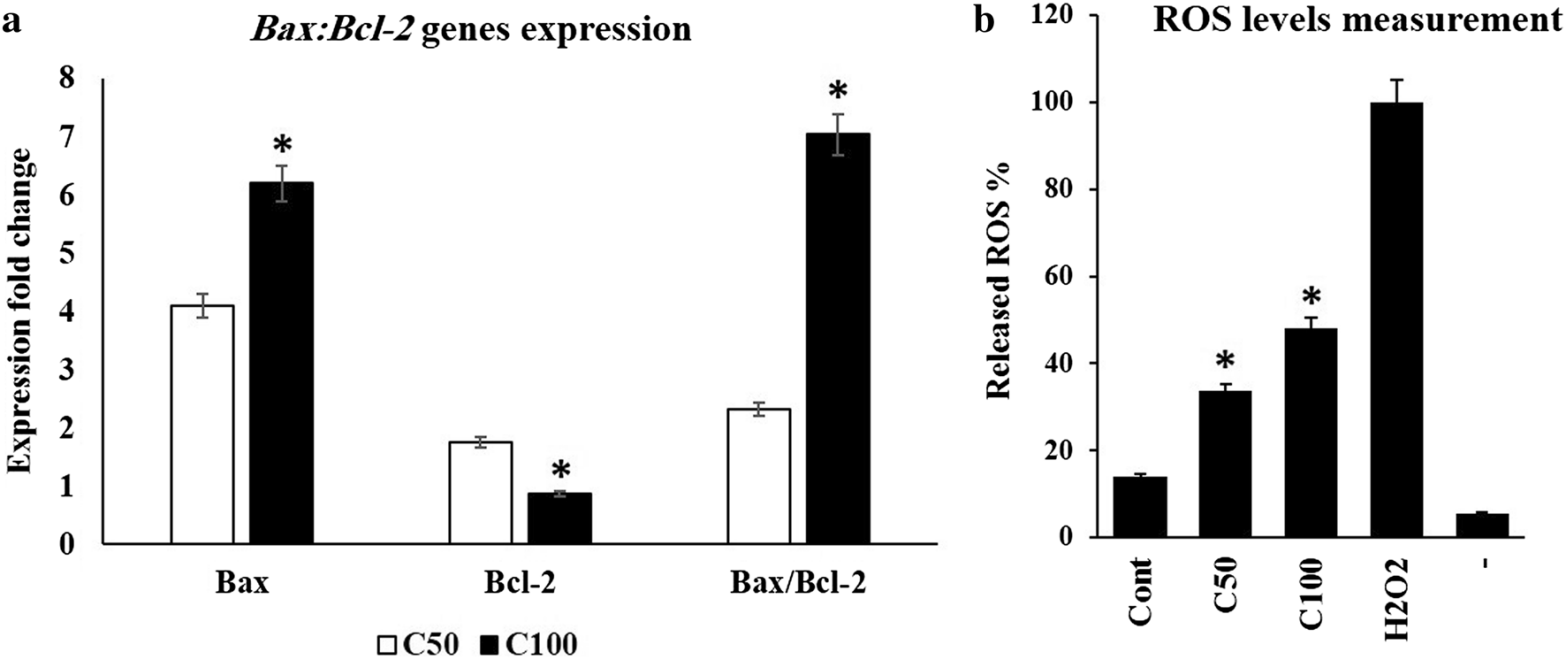
Analysis of mitochondrial apoptotic molecules in treated MDA-MB-231 cells. Untreated (Cont) or treated cells with ASPE at 50 µg/mL (C50) and 100 µg/mL (C100). **a** Comparison of change in expression of *Bax* and *Bcl*-*2* genes is mentioned in* diagrams* as fold change (ratio of target/reference gene) in MDA-MB-231 cells. Each* data point* represents the mean (±SEM) of three independent experiments. **b** ROS measurement by Amplex Red™. Cells were treated with different concentrations of ASPE and incubated for 12 h. The difference between absorbance for treated and untreated cells samples was measured with a fluorescence-based microplate reader using excitation at 530 ± 12.5 nm and fluorescence detection at 580 ± 25 nm. The determined for a no-H_2_O_2_ control reaction, was subtracted from each value. The* Graph* demonstrates the percentage of H_2_O_2_ from Amplex Red assay based on mean absorbance. Each* data point* in all diagrams is representing the mean (±SEM) of three independent experiments. *Asterisks* indicate significant difference (*P* < 0.05)

We also followed up the ASPE signalling by inspecting the expression of mitochondrial Bax (pro-apoptotic) and Bcl-2 (anti-apoptotic) related to apoptosis induction at 50 and 100 µg/mL. Here, we showed that ASPE induces a significant misbalance of *Bax:Bcl*-*2* transcripts ratio of ASPE-treated cells by up-regulation of *Bax* gene to reach 4.1-fold and down-regulation of *Bcl*-*2* gene to reach 1.76-fold (Fig. 6a). In the other hand, the effect of treatment of MDA-MB-231 cells with ASPE on the induced levels of ROS was inspected by using Amplex Red™ assay. ROS concentration was determined in treated and lysed cells to measure the total ROS released by the cells. In this assay, cells treated with 50 and 100 µg/mL ASPE displayed a marked increase in the levels of total ROS levels compared to the untreated control cells. ASPE was able to increase the ROS signal depending on concentrations tested (Fig. 6b). Therefore, these data suggest that the disruption of mitochondria function by the misbalance of Bax:Bcl-2 ratio and ROS induction are involved in the ASPE-induced apoptosis of MDA-MB-231 cells.

## Discussion

Algal sulfated polysaccharides have attracted more attention due to their immune modulatory and anti-tumor properties [26–28]. Seaweed polysaccharides are presented by alginates, agars, carrageenans, ulvanes, and fucoidans, which are widely used in the food and pharmaceutical industry and also in other branches of industry [29].

Several recent studies have illustrated the anti-proliferative effect of polysaccharides deviated from difference resources. Polysaccharides from *Tupistrachinensis* induced severe apoptosis in a cancerous tissue in H22 hepatocarcinoma mice animal model [30]. Other study revealed that, the polymeric black tea polyphenols modulate TAP-induced molecular and biochemical alterations in mouse skin like the activation of transcription factors related to cell proliferation, apoptosis and inflammation [31]. Although, fucoidan (sulfated polysaccharide obtained from brown seaweeds) induced apoptosis, inhibited angiogenesis and suppressed lung metastasis of breast cancer in 4T1 mouse breast cancer cells and in BALB/c mice bearing breast cancer [32]. Even though, sulfated polysaccharide fraction from the brown alga Laminaria japonicacan effectively inhibited the proliferation of cervical carcinoma U14 cells in vitro, and could not only significantly inhibited the growth of U14 implanted tumor but also induced apoptosis of tumor tissue in tumor-bearing mice [33].

**Fig. 7:**
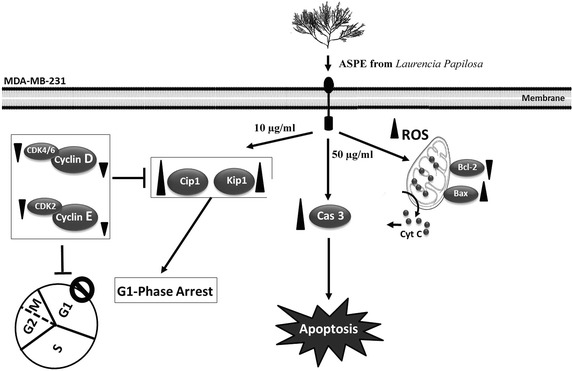
Proposed pathway of ASPE signaling in MDA-MB-231 cells.* Diagram* indicates: Induction of G1-phase cell cycle arrest and apoptosis in MDA-MB-231 cells. Low dose (10 µg/ml) of ASPE induce G1-phase arrest accompanied by up-regulation of *Cip1/p21* and *Kip1/p27* and down-regulation of cyclin *D*, and cyclin *E* transcripts and their related inhibitors *Cdk2*, *Cdk4*, and *Cdk6*.High dose (50 µg/ml) induce apoptosis in MDA-MB-231 cells and increase the expression of cleaved Caspase-3and *Bax*, and decrease the anti-apoptosis protein level of Bcl-2. Over-generation of reactive oxygen species *ROS* was also illustrated.* Black triangles* indicate up-regulation or down-regulation of gene/protein expression where the amplitude of* triangle* illustrates the fold change of expression

Our results showed that ASPE from red seaweed suppresses cell proliferation of MDA-MB-231 cells and arrest them at G1-phase at low dose (10 µg/ml). ASPE also triggers apoptosis in these cells at higher doses (30–50 µg/ml), possibly through enhanced expression of *Bax*, and inhabitation of Bcl-2 protein. The increased ratio of Bax/Bcl-2 and the activation of Caspase-3 in addition to ROS induction are perceptible indicators for such pathway (Fig. 7).

The same behaviour of treated-MDA-MB-231/Her-2 cancers cells with curcumin (a hydrophobic polyphenol derived from the plant, *curcuma longa*) was observed depending on the concentration of the applied treatment. This compound induces G1-phase arrest at a 30 μM whereas, it triggers apoptosis at 50 μM and blocks cell migration. A low dose of curcumin cause increases p27 and decreases Skp2, Her2, Cyclin E, CDK kinases in a time and dose-dependent manner. However, higher doses of curcumin initiate a dose-dependent apoptotic death in MDA-MB-231 by cleaving forms of PARP and Caspase-3 [34].

Importantly, control of cell cycle progression in cancer cells is considered to be a potentially effective strategy for the control of tumor growth [35]. The molecular analyses of human cancers have revealed that cell cycle regulators are frequently mutated in most common malignancies [36]. Our data indicated that treatment of MDA-MB-231 cells at 10 µg/mL ASPE resulted in significant G1-phase arrest of cell cycle progression, which indicates that one of the mechanisms by which ASPE may act to inhibit the proliferation of cancer cells is inhibition of cell cycle progression.

Many other compounds have the same effects as ASPE on MDA-MB-231 cells. Boehmeriasin A isolated from *Boehmeria siamensis* Craib for example considered as proliferation inhibitor of MDA-MB-231 via G1 phase cell cycle arrest. It also induced differentiation in those cells [37]. Physcion (anthraquinone from *rhubarb*) is another example. This compound also has anti-proliferative effects on MDA-MB-231 mediated by inducing G0/G1 phase arrest [38].On the other hand, both fractions (pentane and pentane/diethyl ether fractions) which isolated from *Daucuscarota* inhibits cell proliferation by inducing cell cycle arrest in MDA-MB-231 cells through the inhibition of the MAPK/ERK pathway [39].

When cells are injured, CDK inhibitory genes are up-regulated then, the G1-phase-related Cyclin-CDK complexes are down-regulated for promoting cell cycle arrest [40]. Our finding of a significant decrease in cyclins *D1*, *D2*, and *E1* and their related inhibitors *Cdk2*, *Cdk4*, and *Cdk6* in MDA-MB-231 cells after treatment with ASPE suggests the disruption of the uncontrolled cell cycle progression of these cells (Fig. 3). Therefore, this result suggests that the ASPE induced G1-phase arrest is mediated through the up-regulation of Cip1/p21 and Kip1/p27 transcripts, which enhances the formation of heterotrimeric complexes with the G1-S Cdks and cyclins thereby inhibiting their activity (Fig. 3). Kip1/p27 is up-regulated in response to anti-proliferative signals [41]. Zhuang et al. demonstrated that treatment of human breast cancer cell lines with Metformin (oral anti-hyperglycemic drug) activated AMPK which caused the loss of cyclin D1 mRNA and downregulation of cyclin D1 protein. The reduction in cyclin D1 resulted in the release of sequestered cell cycle inhibitors Kip1/p27 and Cip1/p21. The released CDK inhibitors bind to and inhibit cyclin E/CDK2, thus preventing cell cycle progression from G1 to S phase [42].

The increased expression of G1 cyclins in cancer cells provides an uncontrolled growth advantage because most of these cells either lack Cdk inhibitors or the expression of Cdk inhibitors is not at a sufficient level to control Cdk-cyclin activity [40]. G1-phase arrest of cell cycle progression provides an opportunity for cells to either undergo repair mechanisms or follow the apoptotic pathway.

The tumor suppressor TP53 plays an important role in response to DNA damage and other genomic instability. Functional TP53 protein is crucial in TP53-dependent pathway leading to cell cycle arrest or apoptosis [43]. MDA-MB-231 cells are known to contain mutated, functionally inactive TP53 [44, 45]. The increase in TP53 protein following ASPE treatment (data not shown) may not solely explain TP53-dependent apoptosis. Therefore, the up-regulation of Cip1/p21 gene, cell cycle arrest and apoptosis in MDA-MB-231 cells is chiefly mediated through a TP53-independent mechanism.

On the other hand, our flow cytometry data indicate that treatment of MDA-MB-231 cells with 25 and 50 µg/mL of ASPE resulted in significant induction of apoptosis (Fig. 4). Apoptosis plays a crucial role in eliminating the mutated neoplastic and hyperproliferating neoplastic cells from the system and therefore is considered as a protective mechanism against cancer progression [46, 47]. Apoptosis has been shown as a significant way of cell death after cytotoxic drug treatment in a variety of cancer types [48]. Therefore, an understanding of apoptosis events and its pathway may allow the development of novel agents for cancer treatment [49]. Nuclear condensation, DNA fragmentation, cell shrinkage and cell membrane disintegration are common apoptotic features [50–52]. Interestingly, our results demonstrated that ASPE effectively induces apoptosis by dose and time dependent manner in MDA-MB-231 cells (Fig. 4). Apoptosis is tightly regulated by anti-apoptotic and proapoptotic effector molecules, including Caspase-3 and Bcl-2 protein family. As the activity of the regulatory molecules can be lost in cancer cells, it is important to elucidate the mechanisms by which anti-apoptotic molecules exert their effects, especially in MDA-MB-231 cells. Thus, we investigated the ASPE mode-of-action and by consequent described the characterized apoptosis induction in ASPE-treated cells. We found that ASPE treatment of MDA-MB-231 cells resulted in a dose-dependent activation of Caspase-3 demonstrated by flow cytometry (Fig. 5). This confirmed the role of Caspase-3 in the ASPE-induced apoptosis.

The Bcl-2 family of proteins is the central regulators of the mitochondrial cell-intrinsic apoptotic [53]. The Bcl-2 itself binds to pro apoptotic members such as Bax, preventing pore formation and cytochrome c release [54–56]. In contrast, increase in expression of Bax, induces cell death eliminating tumor cells [57–59]. Therefore, we investigated the contribution of Bcl-2 family proteins to ASPE-induced apoptosis of MDA-MB-231 cells. We found that treatment of MDA-MB-231 cells with ASPE resulted in a pronounced increase in the expression of *Bax* transcripts and a decrease in the transcripts expression of *Bcl*-*2* (Fig. 6a). Consequently, we confirmed the decrease of Bcl-2 protein after ASPE treatment by flow cytometry (Fig. 5b). This misbalance may be responsible for the concomitant execution phase of apoptosis that we observed, which included disruption of mitochondria functionality. ROS generation in apoptosis induction by some agents has been shown to occur downs mitochondrial disruption [60, 61]. Our results also showed that ASPE induced ROS in a dose-dependent manner (Fig. 6b). ROS may as well participate in apoptosis induced by ASPE. Similar results were observed in human umbilical vein endothelial cells (HUVECs) exposed to high concentration of λ-carrageenan oligosaccharides (λ-CO) which activated the mitochondrial-mediated apoptotic pathway and triggered ROS production [62]. Overviewing our data together, the biological activity of ASPE algal extract demonstrates a potent mechanism for cell cycle arrest and apoptosis induction. These results are conformed to numerous studies mentioning cytostatic effects within algal sulfated polysaccharides treatment [63, 64].

## Conclusions

The results of this study do support a pervious study about the role of algal sulphated polysaccharidic extract ASPE in inducing cell cycle arrest and apoptosis induction in MDA-MB-231 breast cancer cells. The ASPE could be a promising target molecule for developing a new anti-cancer drugs. Nevertheless, further studies are warranted to evaluate its potential anti-proliferative and anti-cancerous activities in vivo.

## Abbreviations

ASPE: algal sulfated polysaccharide extract
SP: sulfated polysaccharide
PI: propidium iodide
SSC: side-scatter light
cDNA: complementary DNA
FITC: fluorescein isothiocyanate
ROS: reactive oxygen species
H2O2: hydrogen peroxide
ANOVA: analysis of variance
CDKs: cyclin-dependent kinases
Cip1: CDK-interacting protein 1
Kip1: kinase interacting protein 1
Bax: (protein coding), Bcl2-associated X protein
